# Characterization and Electrochemical Properties of Porous NiCo_2_O_4_ Nanostructured Materials Synthesized Using an In Situ Polymerization Template Method

**DOI:** 10.3390/ma19030458

**Published:** 2026-01-23

**Authors:** Chunyang Li, Changsheng An, Guojun Li

**Affiliations:** 1 Metal-Oxygen New Energy Batteries Key Laboratory of Liaoning Province, Dalian Jiaotong University, Dalian 116028, China; dl_licy@126.com; 2 Hunan Key Laboratory of Applied Environmental Photocatalysis, Changsha University, Changsha 410022, China; z20190628@ccsu.edu.cn

**Keywords:** NiCo_2_O_4_, porous nanostructures, electrochemical properties, polymerizing template method, supercapacitor

## Abstract

**Highlights:**

**What are the main findings?**

- Porous NiCo_2_O_4_ nanomaterials were prepared by using an in situ synthesized template method.
- Porous NiCo_2_O_4_ exhibits a 3D macroporous/mesoporous structure.
- Porous NiCo_2_O_4_ nanomaterials are advantageous for increasing cycle life and high-rate performance.

**What are the implication of the main findings?**

- The electrode material should be designed with a porous structure.
- It is necessary to adopt appropriate preparation methods.

**Abstract:**

Porous NiCo_2_O_4_ nanomaterials were synthesized using in situ-generated polyacrylamide as a template, with cobalt nitrate, nickel nitrate, and urea serving as raw materials. XRD and FESEM analyses confirm the successful formation of spinel-structured NiCo_2_O_4_ electrode materials featuring a 3D macroporous/mesoporous architecture and an average crystalline size of approximately 8.1 nm, obtained through calcination of the amorphous precursor. Electrochemical evaluation of the as-prepared NiCo_2_O_4_ reveals that the specific capacitance retained at 10 A g^−1^ reaches 88.9% of the value measured at 1 A g^−1^, demonstrating excellent rate capability. Furthermore, the material exhibits a gradual increase in specific capacity over 3000 charge–discharge cycles, achieving a capacitance retention of up to 246.5%, which indicates good cycling stability and superior capacity retention.

## 1. Introduction

Spinel-type NiCo_2_O_4_ has emerged as a highly promising electrode material for pseudocapacitor supercapacitors [1,2] and lithium-ion battery anodes [3,4], owing to its favorable attributes such as low toxicity, abundant natural resources, high theoretical capacity, and excellent redox reversibility. However, for practical applications, the electrical conductivity, specific capacitance, rate capability, and cycling stability of NiCo_2_O_4_ still require further enhancement. In particular, rate performance and long-term cycle stability are critical factors that determine its applicability. To date, several effective strategies have been employed, including the construction of porous structures [1,2,3], material compositing [5,6,7,8], and the design of nanostructures with specific morphologies, such as one-dimensional (1D) nanowires and nanoneedles [9,10], two-dimensional (2D) nanosheets [11,12], and three-dimensional (3D) nanoflowers and nanocabbage [13,14] or nanospheres [15]. To achieve more optimal electrochemical performance, combining two or more of these methods is often necessary [16,17,18]. Among these strategies, introducing porosity has been shown to significantly improve the cycling stability and service life of electrode materials during repeated charge–discharge processes. Generally, there are primarily two approaches currently used for the synthesis of porous NiCo_2_O_4_ materials. The first approach is the template method. This method typically employs conductive Ni foam [12,16,19] and carbon-based materials as templates, where the carbon materials may include carbon textiles [20,21], or heat-treated biomass-derived porous carbon such as mollusc shell [22], pomelo peel [23], etc. By combining these with a hydrothermal process, porous nickel cobaltate with one-dimensional (1D), two-dimensional (2D), and three-dimensional (3D) tailored morphologies can be synthesized. The resulting NiCo_2_O_4_ materials exhibit high specific capacitance and favorable rate performance; however, this method incurs relatively high costs when scaling up for batch production. The second approach is the particle aggregation method, which primarily involves techniques such as spray drying [24], co-precipitation [25], and the sol–gel process [26,27]. In this method, mesopores are formed through the aggregation and growth of nanoparticles during the calcination process. While this approach is cost-effective and easily scalable, the electrochemical performance of the resulting materials is generally inferior. Gelcasting was initially developed for ceramic shaping [28] and has since been adapted for the preparation of ceramic powders [29] based on its underlying principles. The core mechanism involves water-soluble monomers and crosslinking agents that undergo in situ polymerization to form a 3D polymeric network under the initiation of a catalyst. This transforms the solution system into a wet gel with certain elasticity, which is then dried to yield a dry gel [28,29]. Compared to the conventional sol–gel method, the resulting gel exhibits higher mechanical strength and elasticity. After calcination, nanosized powders are obtained. However, due to the high-temperature processing involved, it is challenging to preserve the original 3D network structure, making the fabrication of 3D porous materials difficult. Nevertheless, given that NiCo_2_O_4_ synthesis typically occurs at relatively low temperatures (250–300 °C) [24,26], there is potential to retain the 3D network structure under optimized conditions. To address this opportunity, the present study aims to synthesize porous NiCo_2_O_4_ nanostructured materials by utilizing in situ polymerization of a 3D structured polymer as a template, followed by comprehensive structural characterization and evaluation of their electrochemical properties.

## 2. Experimental

### 2.1. Preparation of the Sample

First, acrylamide (AM, Kermel Chemical Reagent, Tianjin, China), serving as the monomer, and N,N-methylenebisacrylamide (MBAM, Kermel Chemical Reagent, Tianjin, China), used as the crosslinker, were mixed in a weight ratio of 19:1 and dissolved in deionized water to prepare a homogeneous solution containing 7.5 wt% acrylamide. Subsequently, cobalt nitrate (Damao Chemical Reagent, Tianjin, China), nickel nitrate (Damao Chemical Reagent, Tianjin, China), and urea (Chemical Reagent, Shenyang, China), in a stoichiometric ratio of 2:1:4, were dissolved in the above-prepared solution to yield 100 mL of a precursor solution with a total metal ion concentration of 0.3 M. Under continuous stirring, 0.2 g of freshly prepared 10 wt% ammonium persulfate (Sinopharm Chemical Reagent, Shanghai, China), used as initiators, were added dropwise into the solution in sequence. Immediately after the addition of initiators, the solution was rapidly transferred into a sealed container. After standing undisturbed for 20 min, in situ polymerization led to the formation of a polyacrylamide wet gel with a three-dimensional (3D) network structure. The sealed container was then placed in an oven (Haoyue Instrument and Equipment, Shanghai, China) at 105 °C for 6 h to facilitate the reaction of cobalt nitrate, nickel nitrate, and urea within the 3D polymeric network. The resulting wet gel was removed and dried at 100 °C for 24 h to obtain the dried gel precursor. This precursor was subsequently calcined in air at 300 °C for 2 h, yielding a black, fluffy product. To remove sulfate ions and other residual soluble species, the calcined sample was thoroughly washed with deionized water until no white precipitate was observed upon testing the filtrate with 0.1 M barium acetate solution (Sinopharm Chemical Reagent, Shanghai, China). Finally, the purified sample was dried at 100 °C for 12 h to obtain the final product.

### 2.2. Material Characterization

X-ray diffraction (XRD) patterns were collected using a Panalytical D/Max-Ultima diffractometer equipped (Malvern Panalytical, Almelo, The Netherlands) with Cu Kα radiation. The morphology and crystal structure were examined via field-emission scanning electron microscopy (FESEM, SUPRA 55, ZEISS, Oberkochen, Germany) and high-resolution transmission electron microscopy (HRTEM, JEM-2100F, JEOL Ltd., Tokyo, Japan). Specific surface area and pore size distribution were determined by N_2_ adsorption–desorption measurements using the BET method (Quantachrome 3SI-MP-11, Quantachrome Instruments, Boynton Beach, FL, USA). Surface elemental composition and chemical states were analyzed by X-ray photoelectron spectroscopy (XPS, PHI 5700, Physical Electronics, Chigasaki, Japan).

### 2.3. Electrochemical Measurements

The working electrode was fabricated by homogeneously mixing 80 wt% synthesized NiCo_2_O_4_, 10 wt% acetylene black, and 10 wt% polyvinylidene fluoride (PVDF) in 1-methyl-2-pyrrolidone (NMP), followed by ultrasonic dispersion to ensure uniformity. The resulting slurry was uniformly coated onto a cleaned nickel foam substrate with an active area of 1 cm^2^ and subsequently dried under vacuum at 110 °C for more than 12 h. The loading mass of the active material on the electrode was approximately 1 mg. For the three-electrode electrochemical measurements conducted using an electrochemical workstation (CHI600B, CH Instruments, Shanghai, China), a Hg/HgO electrode served as the reference electrode, a platinum electrode was employed as the counter electrode, and a 2 M KOH aqueous solution was used as the electrolyte.

## 3. Results and Discussion

### 3.1. Characterization of As-Calcined NiCo_2_O_4_

**Figure 1:**
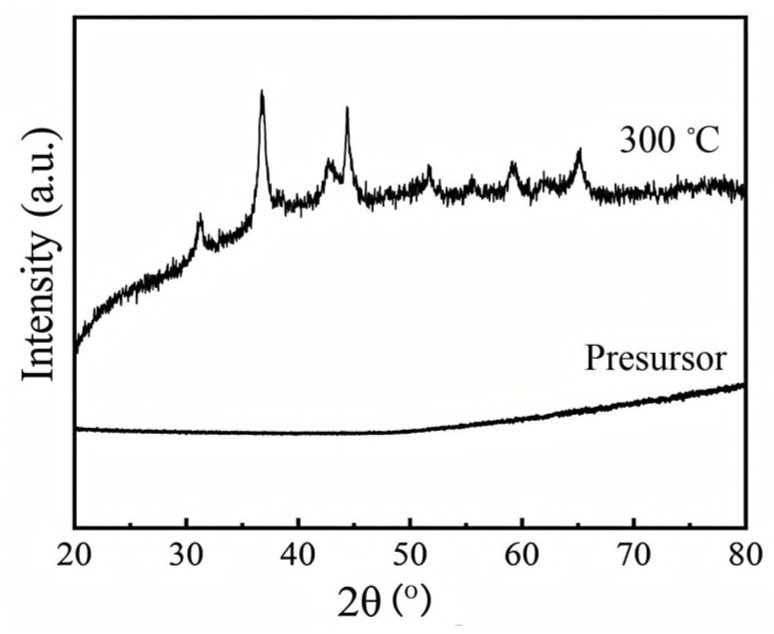
XRD pattern of the precursor and as-prepared NiCo_2_O_4_.

Figure 1 presents the XRD patterns of the precursor and the as-calcined NiCo_2_O_4_. As shown in Figure 1, no distinct diffraction peaks are observed in the precursor, indicating its amorphous nature. In contrast, the XRD pattern of the calcined NiCo_2_O_4_ exhibits well-defined diffraction peaks at 2θ angles of 31.4°, 36.75°, 44.66°, 59.06°, and 65.01°, which can be indexed to the (220), (311), (400), (511), and (440) crystal planes, respectively, of the spinel-type NiCo_2_O_4_ referenced to the standard JCPDS card No. 73-1702. These results confirm the successful formation of a spinel-phase NiCo_2_O_4_ structure upon calcination at 300 °C. In addition, the diffraction peak has an obvious broadening effect, suggesting that the particles are very fine.

**Figure 2:**
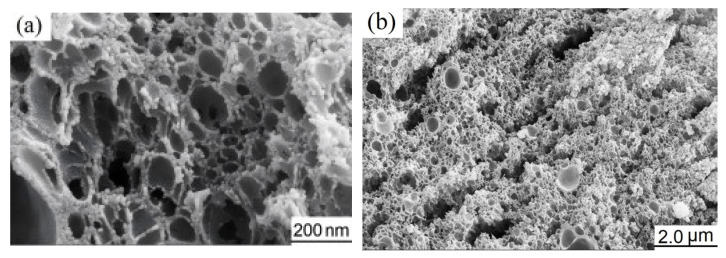
FESEM images of as-synthesized NiCo_2_O_4_. (**a**) 50k×, (**b**) 10k×.

Figure 2 is FESEM images of as-prepared NiCo_2_O_4_. It can be observed from the images that the NiCo_2_O_4_ exhibits a porous structure; the majority of the pores are smaller than 200 nm in size, and a few can reach 1 μm. The formation of macropores is caused by the thermal decomposition and expansion of the in situ synthesized polyacrylamide polymer with a 3D network and the produced inorganic compounds during the calcination.

**Figure 3:**
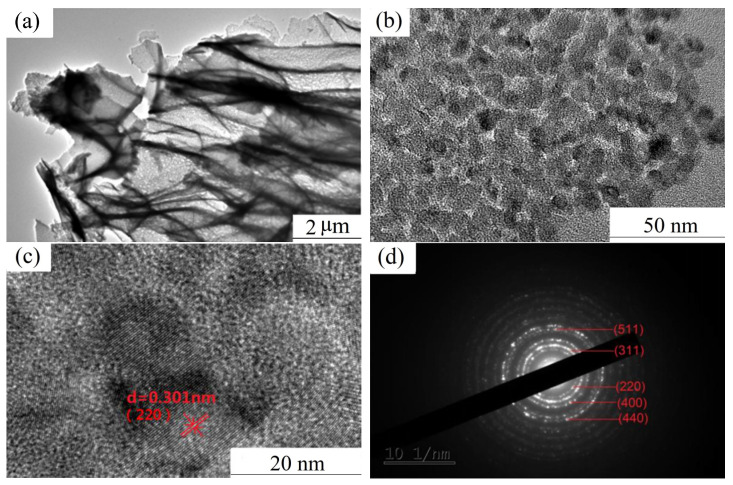
TEM and SAED images of as-calcined NiCo_2_O_4_. (**a**,**b**) TEM images with different magnifications. (**c**) HRTEM. (**d**) SAED image.

To gain deeper insights into the pore wall structure, TEM was utilized to examine the microstructure of the as-calcined samples. Figure 3 presents the TEM and selected-area electron diffraction (SAED) images of the as-calcined NiCo_2_O_4_. As shown in Figure 3a, the pore walls are extremely thin, approximately equivalent to the thickness of a single nanocrystalline grain. The relatively thick dark contrast regions are attributed to the molecular chains of the polymer network. The grain size (Figure 3b) exhibits a narrow distribution, averaging ~8.1 nm, and uniformly sized nanopores are observed at the grain boundaries. These observations indicate that the 3D polymer network structure effectively suppresses abnormal grain growth and enables the formation of uniform, nanostructured pores. HRTEM (Figure 3c) and SAED (Figure 3d) reveal that NiCo_2_O_4_ is polycrystalline. The interplanar spacings and diffraction patterns indexed to the space group Fd-3m, as determined by Digital Micrograph software 5.0, are in good agreement with the corresponding data for spinel-type NiCo_2_O_4_ (JCPDS card No. 20-0781). These characterizations further confirm the formation of spinel-structured NiCo_2_O_4_.

**Figure 4:**
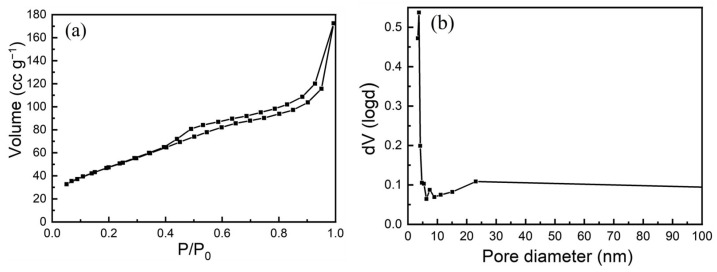
N_2_ adsorption and desorption isotherms of the calcined sample. (**a**) BET, (**b**) BJH.

Figure 4 presents the N_2_ adsorption–desorption isotherm results. As shown in Figure 4a, the curve exhibits a typical Type IV isotherm, with a distinct hysteresis loop observed in the relative pressure (P/P_0_) range of 0.4–1.0, indicating the presence of mesoporous structures. According to the Barrett–Joyner–Halenda (BJH) pore size distribution analysis (Figure 4b), the pores are predominantly small, with an average diameter of approximately 3.74 nm, and display a narrow size distribution, which can be attributed to the uniformity of the pore wall structure. The specific surface area of the sample is determined to be 76.84 m^2^ g^−1^, suggesting that the as-calcined NiCo_2_O_4_ particles are of nanoscale dimensions. These structural characteristics are favorable for efficient charge transfer and electrolyte ion diffusion.

**Figure 5:**
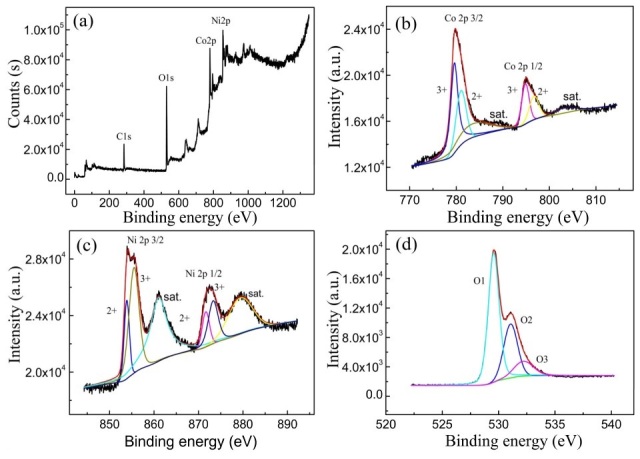
XPS of the NiCo_2_O_4_ sample. (**a**) XPS spectrum. (**b**) Ni 2p, (**c**) Co 2p, (**d**) O 1s.

To further characterize the elemental composition and oxidation states in NiCo_2_O_4_, XPS analysis was performed on the sample, with the resulting spectra presented in Figure 5. The survey spectrum (Figure 5a) confirms the presence of Ni, Co, and O, along with a minor carbon signal used as a charge reference, while no detectable impurities are observed. The core-level XPS spectra were deconvoluted using a Gauss–Lorentz fitting method. The Ni 2p spectrum (Figure 5b) displays two distinct spin–orbit doublets corresponding to Ni^2+^ and Ni^3+^, accompanied by two shake-up satellite peaks (labeled “sat.”). The binding energies at 854.79 eV and 871.62 eV are assigned to Ni^2+^, whereas those at 855.55 eV and 873.35 eV are attributed to Ni^3+^ [3,30]. Similarly, the Co 2p spectrum (Figure 5c) exhibits characteristic spin–orbit doublets for Co 2p_3_/_2_ (779.80 eV) and Co 2p_1_/_2_ (795.05 eV), along with two satellite peaks (sat.). Peaks at 781.10 eV and 796.80 eV are associated with Co^2+^, while the binding energies at 779.70 eV and 794.90 eV correspond to Co^3+^ [1,31]. The O 1s spectrum (Figure 5d) reveals three oxygen species: O 1 (529.54 eV) is assigned to lattice oxygen in metal–oxygen bonds, O 2 (531.8 eV) corresponds to oxygen vacancies or low-coordinated oxygen sites in the nanostructured material, and O 3 (532.2 eV) is likely due to surface-adsorbed water molecules, either physically or chemically bound [32]. These findings collectively confirm the coexistence of Ni^2+^, Ni^3+^, Co^2+^, and Co^3+^ in the NiCo_2_O_4_ nanostructure, consistent with previously reported results for NiCo_2_O_4_ materials [33].

### 3.2. Electrochemical Properties

**Figure 6:**
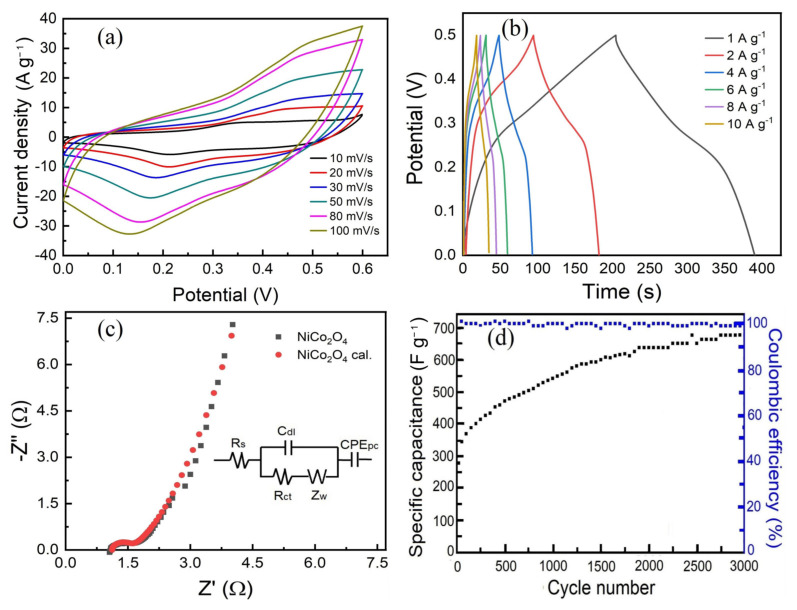
Electrochemical properties of as-calcined NiCo_2_O_4_. (**a**) CV curves at different scan rates, (**b**) GCD curves at different current densities, (**c**) EIS curve, and (**d**) cycle life and Coulomb efficiency of as-prepared NiCo_2_O_4_ nanomaterial at 10 A g^−1^.

Figure 6 presents the electrochemical performance of as-calcined NiCo_2_O_4_. Figure 6a displays the cyclic voltammetry (CV) curves recorded at various scan rates. In contrast to the nearly rectangular CV profile characteristic of double-layer capacitance [1], the CV curves of the porous NiCo_2_O_4_ nano-electrode material exhibit a typical pseudocapacitive behavior. Within the voltage window of 0–0.6 V (vs. Hg/HgO), a pair of symmetric redox peaks is observed on each curve, indicating that charge storage is governed by reversible Faradaic redox reactions involving Co^2+^/Co^3+^ and Ni^2+^/Ni^3+^ couples. Equations (1) and (2) [34,35] can be used for redox reactions:

(1) $$Ni{Co}_{2}O_{4}+OH^{-}+H_{2}O⟷NiOOH+2CoOOH+2e^{-}$$

(2) $$CoOOH+OH^{-}⟷CoO_{2}+H_{2}O+e^{-}$$

The CV curves reveal that the enclosed area increases with rising scan rate, while the oxidation and reduction peaks shift toward higher and lower potentials, respectively. Specifically, the oxidation peak potential shifts from approximately 0.35 V to 0.5 V, and the reduction peak potential shifts from around 0.18 V to 0.13 V. With increasing scan rate, the separation between the anodic and cathodic peak potentials enlarges, suggesting the presence of electrode polarization. Nevertheless, the overall shape of the CV curves is well-retained across all scan rates, demonstrating good structural integrity and cycling stability of the electrode material.

Figure 6b shows the galvanostatic charge–discharge (GCD) curves of porous NiCo_2_O_4_ nano-electrode materials at different current densities within the voltage range of 0–0.5 V. The specific capacitance was calculated by the following Equation (3).

(3) $$C_{s}=I∆t/m∆V$$

where *C_S_* (F g^−1^) is the specific capacitance, *I* (A) is the discharge current, ∆*t* (s) is the discharge time, ∆*V* (V) represents the potential window and m (g) represents the mass of active material. The specific capacitance calculated from discharge time according to Equation (3) is 371.2, 362.5, 356, 358.67, 344.4, 340.8 and 330.0 F g^−1^ corresponding to the discharging current densities of 1, 2, 4, 6, 8 and 10 A g^−1^, respectively. The specific capacitance gradually decreased at higher current density due to the incremental voltage drop and the insufficient active material involved in the redox reaction at higher current densities. However, the as-fabricated NiCo_2_O_4_ exhibits a good rate capability at all the current densities evaluated. Even at the current density of 10 A g^−1^, the specific capacitance still remains 88.9% of the initial capacitance. Figure 6c presents the electrochemical impedance spectroscopy (EIS) results of the nanostructured porous NiCo_2_O_4_, along with the corresponding fitting plot derived from the equivalent circuit model. As shown, the equivalent circuit comprises the following components: *Rs*, *Rct*, *Z_W_*, *C_dl_*, and *CPE_PC_* [36]. *Rs* represents the combined solution resistance and contact resistance [37,38], which is determined from the intercept of the high-frequency region of the Nyquist plot with the real axis. *Rct* denotes the charge transfer resistance associated with Faradaic reactions and corresponds to the diameter of the semicircular arc observed in the medium-to-high frequency range. The Warburg impedance (*Z_W_*) reflects the diffusion resistance of OH^−^ ions within the active material particles, as indicated by the inclined line in the low-frequency region. *C_dl_* and CPEPC refer to the electric double-layer capacitance at the electrode–electrolyte interface and the constant phase element, respectively [38]. In comparison with data from the literature [39], the synthesized sample exhibits a lower *Rct* value of 0.65 Ω than that of 0.75 Ω, indicating favorable conditions for rapid charge transfer. Furthermore, the steep slope of the low-frequency linear region suggests a high diffusion coefficient for OH^−^ ions. The efficient transport of both charges and ions can be attributed to the abundant macroporous and mesoporous architecture, as well as the minimal resistance resulting from the ultrafine grain structure. Consequently, the fabricated porous NiCo_2_O_4_ nanomaterial demonstrates enhanced electrochemical reversibility.

For supercapacitors, the variation in capacitance during prolonged operation at high current densities represents a critical electrochemical performance metric for practical applications. Figure 6d presents the cycling stability and Coulombic efficiency of porous NiCo_2_O_4_ nanostructured electrode materials tested at a current density of 10 A g^−1^. The specific capacitance increases progressively with cycle number, rising from an initial value of 275.2 F g^−1^ to 678.4 F g^−1^ after 3000 cycles, corresponding to capacitance retention of 246.5%, indicating good capacity sustainability. During the first 1000 cycles, the capacitance exhibits a rapid increase, which can be attributed to the gradual activation of the active material. This activation enhances the interfacial contact between the electrolyte and the electrode, promotes redox reactions, and is further facilitated by the material’s porous architecture, which reduces charge transfer resistance and supports efficient electrolyte penetration and ion diffusion throughout repeated charge–discharge processes. Beyond 1500 cycles, the rate of capacitance increase slows, eventually reaching a stable plateau. Notably, the Coulombic efficiency remains above 99% throughout the test, demonstrating high electrochemical reversibility and outstanding cycling stability. This behavior is primarily due to the large specific surface area, which provides abundant active sites for Faradaic reactions, and the well-developed porous structure, which not only lowers charge transfer resistance but also effectively accommodates redox-induced lattice volume changes during the repeated charge–discharge processes, thereby preserving structural integrity over extended cycling.

## 4. Conclusions

The macroporous/mesoporous NiCo_2_O_4_ nano-electrode materials were successfully synthesized via an in situ polymerization template method at a calcination temperature of 300 °C. The majority of the 3D macropores exhibit sizes below 200 nm, while mesopores located on the pore walls are approximately 4 nm in diameter, with an average grain size of about 8.1 nm. Electrochemical evaluation of the as-prepared NiCo_2_O_4_ reveals that the porous nano-electrode materials possess better rate capability (retaining 88.9% of the specific capacitance at 10 A g^−1^ relative to that at 1 A g^−1^), good cycling stability at high current densities, and remarkable capacity retention (reaching 246.5% of the initial capacity after 3000 cycles). The superior rate performance, high Coulombic efficiency during charge–discharge processes, extended cycle life, and enhanced capacity retention can be attributed to the large specific surface area, which increases the number of active reaction sites, as well as the well-developed macroporous/mesoporous channel architecture that facilitates reduced charge transfer resistance and effectively buffers lattice stress induced by volume changes during repeated charge–discharge cycles.

## Data Availability

The original contributions presented in this study are included in the article. Further inquiries can be directed to the corresponding author.
